# In Vitro Biotransformation of Two Human CYP3A Probe Substrates and Their Inhibition during Early Zebrafish Development

**DOI:** 10.3390/ijms18010217

**Published:** 2017-01-22

**Authors:** Evy Verbueken, Derek Alsop, Moayad A. Saad, Casper Pype, Els M. Van Peer, Christophe R. Casteleyn, Chris J. Van Ginneken, Joanna Wilson, Steven J. Van Cruchten

**Affiliations:** 1 Applied Veterinary Morphology, Department of Veterinary Sciences, University of Antwerp, 2610 Wilrijk, Antwerp, Belgium; evy.verbueken@uantwerpen.be (E.V.); moayad.saad@student.uantwerpen.be (M.A.S.); casper.pype@uantwerpen.be (C.P.); els.vanpeer@uantwerpen.be (E.M.V.P.); Christophe.Casteleyn@UGent.be (C.R.C.); chris.vanginneken@uantwerpen.be (C.J.V.G.); 2 Wilson Tox Lab, Department of Biology, McMaster University, 1280 Main Street West, Hamilton, ON L8S 4K1, Canada; derekrxr@gmail.com (D.A.); wilsojo@mcmaster.ca (J.W.)

**Keywords:** zebrafish, embryo, development, cytochrome P450, activity, in vitro, and biotransformation

## Abstract

At present, the zebrafish embryo is increasingly used as an alternative animal model to screen for developmental toxicity after exposure to xenobiotics. Since zebrafish embryos depend on their own drug-metabolizing capacity, knowledge of their intrinsic biotransformation is pivotal in order to correctly interpret the outcome of teratogenicity assays. Therefore, the aim of this in vitro study was to assess the activity of cytochrome P450 (CYP)—a group of drug-metabolizing enzymes—in microsomes from whole zebrafish embryos (ZEM) of 5, 24, 48, 72, 96 and 120 h post-fertilization (hpf) by means of a mammalian CYP substrate, i.e., benzyloxy-methyl-resorufin (BOMR). The same CYP activity assays were performed in adult zebrafish liver microsomes (ZLM) to serve as a reference for the embryos. In addition, activity assays with the human CYP3A4-specific Luciferin isopropyl acetal (Luciferin-IPA) as well as inhibition studies with ketoconazole and CYP3cide were carried out to identify CYP activity in ZLM. In the present study, biotransformation of BOMR was detected at 72 and 96 hpf; however, metabolite formation was low compared with ZLM. Furthermore, Luciferin-IPA was not metabolized by the zebrafish. In conclusion, the capacity of intrinsic biotransformation in zebrafish embryos appears to be lacking during a major part of organogenesis.

## 1. Introduction

The zebrafish (*Danio rerio*) embryo has emerged as an alternative animal model for developmental toxicity—also called teratogenicity—screening of new drugs and environmental pollutants (reviewed in [1,2,3,4]). The widespread use of the zebrafish is mainly due to its many advantages such as its short generation time and high fecundity resulting in 100–200 eggs per mating, which makes the use of zebrafish embryos less time-consuming in comparison with in vivo mammalian developmental toxicity studies [5]. Moreover, zebrafish embryos and larvae can be used in medium—or high—throughput screening because of their small size (0.5–4 mm) [6] and, as the embryos can be kept in small volumes (100 µL), only a small amount of compound is required (reviewed in [2]). The latter is very useful during early drug development when the availability of a new chemical entity (NCE)—as defined by the Food and Drug Administration (FDA) [7]—is still very low. Additionally, the legislation of the European Union concerning animal experimentation does not consider it to be a test animal until 120 h post-fertilization (hpf), i.e., the stage of independently feeding [8,9]. Zebrafish embryos develop ex utero and the embryos as well as their chorion are optically transparent, which makes them suitable for microscopic observation of (internal organ) malformations at different developmental time points [5,6]. The lack of a maternal barrier during zebrafish development implies direct exposure of the embryo to the parent compound in teratogenicity assays, while mammalian embryos/fetuses are exposed to the parent compound and its metabolites due to drug metabolism by, predominantly, the dam’s liver. Hence, zebrafish embryos depend on their own drug-metabolizing capacity for detoxification and/or bioactivation of xenobiotics. The latter is particularly important for compounds that require bioactivation to exert their teratogenic potential, i.e., so-called proteratogens. A lack of intrinsic biotransformation in the zebrafish embryo can lead to false negative results in teratogenicity assays as proteratogens will be missed. Since drug metabolism, i.e., phase I (mainly oxidation) (reviewed in [10]) and phase II (conjugation) metabolism (reviewed in [11]), in human embryos and fetuses was shown to be immature, the drug-metabolizing capacity of zebrafish embryos during early development is expected to be negligible as well. In addition, the zebrafish liver and intestine—two important drug-metabolizing organs—develop late in organogenesis, i.e., between 72 and 96 hpf, which supports our hypothesis concerning the lack of intrinsic biotransformation by zebrafish embryos. This hypothesis cannot be tested by just exposing zebrafish embryos to known mammalian proteratogens, as has been done previously [12], because this in vivo approach does not distinguish between teratogenic effects caused by the parent compound or by its metabolite. For in vivo studies, also other pharmacokinetic factors besides metabolism, such as absorption, distribution and excretion, may determine the exposure in the zebrafish embryo and thus also the teratogenic outcome. In the present in vitro study, these confounding factors were excluded by focusing only on the intrinsic metabolizing capacity of zebrafish (embryos). For this purpose, we used microsomes—subcellular fractions of endoplasmic reticulum containing cytochrome P450 (CYP) isoenzymes—from whole embryo homogenates and from adult zebrafish livers.

CYP enzymes represent a superfamily of hemoproteins from which the CYP1, CYP2 and CYP3 families are mainly involved in the oxidative metabolism of xenobiotics in man (reviewed in [13,14]). Furthermore, the human CYP3A subfamily metabolizes approximately 50% of drugs that undergo oxidative biotransformation (Table 1) (reviewed in [15]). CYP-mediated drug-metabolism predominantly occurs in the liver, whereas other tissues such as the intestine, brain, lung, kidney, skin, gonads, etc., contribute to a smaller extent (reviewed in [15,16]). Goldstone and colleagues [17] were able to identify the full suite of *CYP* genes in zebrafish and suggested that also in adult zebrafish the CYP families 1–3 and, to a lesser extent, CYP4s are involved in the biotransformation of xenobiotics. Nevertheless, zebrafish *CYP3A* genes do not phylogenetically cluster with mammalian *CYP3A* genes, so differences in CYP3A activity between zebrafish and mammals can be expected [18,19]. Besides the identification of CYPs in adult zebrafish, Goldstone et al. also demonstrated distinct temporal patterns of CYP expression over the course of zebrafish development [17]. In addition to the research of Goldstone et al., other in vitro and in vivo studies have already been performed on the expression and activity of CYP1 and, to a lesser extent, CYP3 enzymes in adult and developing zebrafish [20,21,22,23,24,25,26,27,28,29,30,31,32,33,34,35]. However, results from these studies are inconclusive and in some cases even contradictory. The latter is most likely due to differences in study design, such as in vitro versus in vivo, using mRNA versus protein versus activity level (induced versus basal CYP activity), other developmental time points, quantitative versus qualitative measurements, other substrates/substrate concentrations, etc. Therefore, the drug-metabolizing capacity of zebrafish embryos still remains a point of debate and requires further investigation.

The aim of the present in vitro drug metabolism study was to assess intrinsic CYP activity in zebrafish embryos of 5–120 hpf and, as a reference for the embryos, in the adult zebrafish liver. The activity assays were performed by means of two mammalian CYP substrates, i.e., benzyloxy-methyl-resorufin (BOMR) (Vivid^®^ CYP450 Screening Kits User Guide 2012) and Luciferin isopropyl acetal (Luciferin-IPA) [36,37], which are supposed to be metabolized by the pharmacologically important CYP3A enzyme. Since the zebrafish liver develops late in organogenesis, microsomes were prepared from the whole embryonic body so as to take all the organs of the developing zebrafish into account. In addition to the activity assays, inhibition studies with the non-specific and concentration-dependent CYP inhibitor ketoconazole [38] and with the CYP3A4-specific inhibitor CYP3cide [39] were carried out in adult zebrafish liver microsomes. The inhibition studies with CYP3cide as well as the activity assays with Luciferin-IPA showed differences between zebrafish and mammalian CYP3A activity, which is in concordance with the phylogenetic difference in *CYP3A* gene expression. Furthermore, the results of the present study support our hypothesis regarding the lack of intrinsic biotransformation by zebrafish embryos as the latter were not able to metabolize BOMR during a major part of organogenesis.

## 2. Results

### 2.1. Benzyloxy-Methyl-Resorufin Assay in Adult Zebrafish Liver Microsomes and in Microsomes from Whole Zebrafish Embryo Homogenates

**Figure 1:**
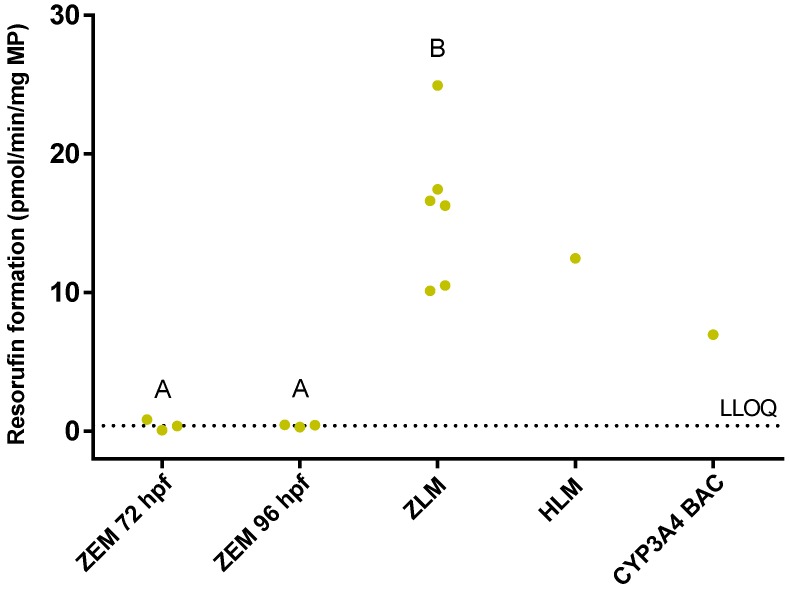
Resorufin formation (pmol/min/mg microsomal protein) by microsomes of zebrafish embryos (ZEM) at 72 and 96 h post-fertilization (hpf) and by liver microsomes from adult female zebrafish (ZLM) after incubation with benzyloxy-methyl-resorufin (BOMR). The dots are the reaction velocities for each batch. Each dot represents the mean value of three technical replicates. The mean reaction velocities for human liver microsomes (HLM) and CYP3A4 Baculosomes^®^ (CYP3A4 BAC) were added to the graph as positive controls. The horizontal dotted line represents the lower limit of quantification (LLOQ). Significant differences (*p* < 0.05) between age groups are indicated by different letters (A and B).

CYP activity was assessed in adult zebrafish liver microsomes (ZLM) and in microsomes from whole zebrafish embryo homogenates (ZEM) of 5–120 hpf by means of the benzyloxy-methyl-resorufin (BOMR) assay. In these experiments, the reaction velocities obtained for ZLM served as a reference for the values of the ZEM. The ZLM were able to convert BOMR into the fluorescent metabolite resorufin, i.e., mean reaction velocity of three technical replicates ± standard deviation (S.D.): 16.28 ± 3.70, 24.95 ± 5.91, 16.63 ± 1.29, 10.52 ± 3.15, 10.12 ± 0.45 and 17.44 ± 1.35 pmol/min/mg microsomal protein (MP) for Batch 1, 2, 3, 4, 5 and 6, respectively (Figure 1). In ZEM, resorufin formation was only observed at 72 and 96 hpf, i.e., 0.42 ± 0.38 pmol/min/mg MP and 0.39 ± 0.09 pmol/min/mg MP, for the respective developmental stages (Figure 1). These latter values were close to the lower limit of quantification (LLOQ) and significantly lower than those of the ZLM (*p* = 0.020 for both comparisons). The reaction velocity of human liver microsomes (HLM) and CYP3A4 Baculosomes^®^ (CYP3A4 BAC) (positive controls) was 12.46 ± 1.41 pmol/min/mg MP and 6.96 ± 1.49 pmol/min/mg MP, respectively.

### 2.2. Inhibition Studies with Adult Zebrafish Liver Microsomes

**Figure 2:**
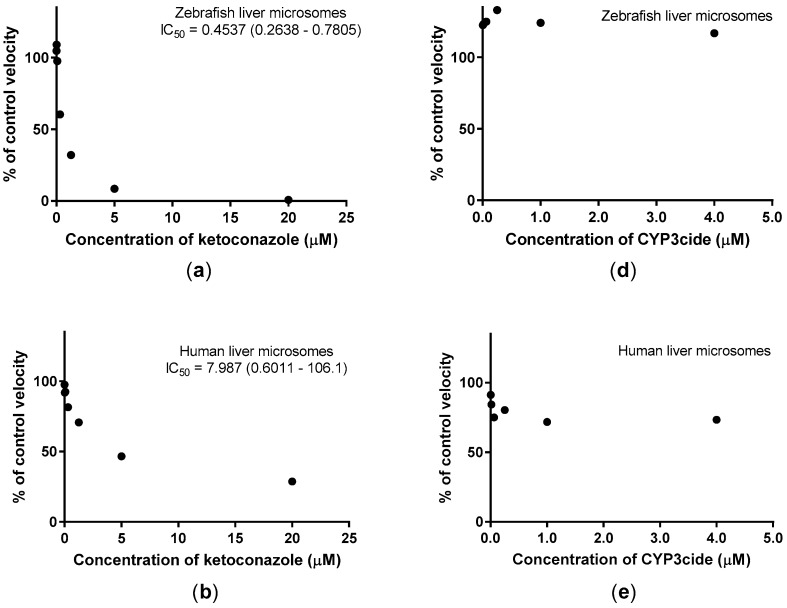

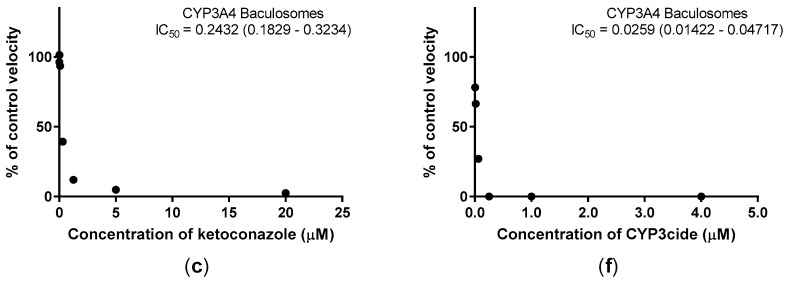
The effect of various concentrations of ketoconazole and CYP3cide on the biotransformation of BOMR. The dots in the graphs represent the percentage ratios of reaction velocity in case of pre-incubation of the microsomes with the respective inhibitor, divided by the control velocity without inhibitor. Graphs (**a**–**c**) show the results for pre-incubation with ketoconazole with (**a**) demonstrating the mean of the results for Batch 1 and Batch 2 of ZLM; whereas (**b**,**c**) show the mean values of the technical replicates with human liver microsomes (HLM) and CYP3A4 Baculosomes^®^ (CYP3A4 BAC), respectively; Graphs (**d**–**f**) show the outcome for pre-incubation with 0–4 µM of CYP3cide (data for 0–2 µM of CYP3cide not shown) with (**d**) representing the mean of the results for Batch 1 and Batch 2 of ZLM; while (**e**,**f**) demonstrate the mean values of the technical replicates with HLM and CYP3A4 BAC, respectively. In case of inhibition, the IC_50_ values and their 95% confidence intervals are added.

Inhibition studies with ketoconazole and CYP3cide were performed in ZLM of Batch 1 and Batch 2 to detect whether these compounds were able to inhibit the biotransformation of BOMR. Figure 2a,d show the mean of the results for Batch 1 and Batch 2 of ZLM. In our study, ketoconazole strongly inhibited the formation of resorufin in ZLM (Figure 2a) and in CYP3A4 BAC (Figure 2c), whereas inhibition of BOMR metabolism was less pronounced in HLM (Figure 2b). In contrast to ketoconazole, CYP3cide did not inhibit the metabolism of BOMR in ZLM (Figure 2d) and inhibition in HLM was limited (Figure 2e). However, CYP3cide strongly inhibited CYP activity in CYP3A4 BAC (Figure 2f). Two ranges of CYP3cide concentrations (0–2 µM and 0–4 µM) were used in our study, showing similar results.

### 2.3. Benzyloxy-Methyl-Resorufin Assay in Cytochrome P450 (CYP) Baculosomes^®^ and in Recombinant Zebrafish CYPs

With the aim of determining whether BOMR is a CYP3A4-specific substrate, CYP Baculosomes^®^ expressing human CYP1A2, CYP2B6, CYP2C9, CYP2C19, CYP2D6 and CYP3A4 were used. These CYP Baculosomes^®^ were selected as they represent the most important CYP enzymes involved in drug metabolism in man (Table 1). The activity assays showed that human CYP2C9, CYP3A4 and in particular CYP1A2 and CYP2B6 enzymes were able to convert the BOMR substrate into the highly fluorescent resorufin (Table 2), whereas no biotransformation of the substrate could be observed for CYP2D6 Baculosomes^®^. Regarding CYP2C19, only two replicates showed values above the LLOQ, while no resorufin was detected in the third replicate. In addition to the human CYP Baculosomes^®^, activity assays with BOMR were performed in recombinant zebrafish CYPs, which showed that the substrate was clearly biotransformed by recombinant CYP1A and to a lesser extent by CYP1B, CYP1C1 and CYP1C2 (Table 2). Resorufin formation was below the LLOQ for CYP1D.

### 2.4. Luciferin-IPA Assay with Adult Zebrafish Liver Microsomes

**Figure 3:**
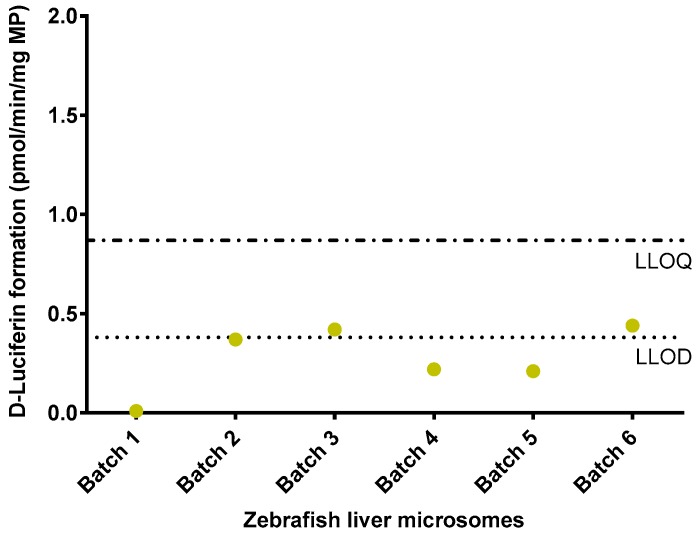
d-Luciferin formation (pmol/min/mg microsomal protein) by liver microsomes from adult female zebrafish. Each dot represents the mean reaction velocity (mean value of three technical replicates) for the corresponding batch of adult zebrafish liver microsomes. The lower horizontal dotted line demonstrates the lower limit of detection (LLOD) and the upper horizontal dash-dotted line represents the lower limit of quantification (LLOQ).

The luminogenic substrate Luciferin-IPA was used to investigate whether ZLM are able to convert this human CYP3A4-specific substrate into d-Luciferin. However, for all batches, metabolite concentrations were below the LLOQ (mean reaction velocity of six batches ± S.D.: 0.28 ± 0.16 pmol/min/mg MP) (Figure 3). In contrast to ZLM, reaction velocities of the positive controls were considerably higher: 463.90 ± 117.28 pmol/min/mg MP and 82.60 ± 43.99 pmol/min/mg MP for HLM and CYP3A4 BAC, respectively. Since no biotransformation of Luciferin-IPA could be observed for ZLM, luminogenic activity assays were not performed in zebrafish embryos.

## 3. Discussion

In view of zebrafish embryos being extensively used in developmental toxicity studies, the present study contributes to a better understanding of the drug-metabolizing capacity of zebrafish embryos. Since CYP enzymes are predominantly involved in the metabolism of xenobiotics, CYP activity assays were performed in zebrafish embryos at different developmental time points and in liver microsomes from adult zebrafish, which served as a reference for the embryos.

The CYP activity assays with BOMR in ZLM showed reaction velocities that were in the same range of those of HLM. This similarity is not surprising as Goldstone et al. [17] identified CYP1, CYP2 and CYP3 families in adult zebrafish and suggested that these enzymes are involved in the biotransformation of xenobiotics as described in humans (reviewed in [15]). Nevertheless, the human CYP3 family only consists of the CYP3A subfamily, from which CYP3A4 plays a predominant role in drug metabolism [40], whereas in zebrafish more CYP3 subfamilies have been characterized, i.e., the CYP3A65 isoform [17,35] and the CYP3C1–3C4 isoforms [22,34]. The *CYP3A65* gene and *CYP3C1* gene were first described by Tseng et al. [35] and Corley-Smith et al. [22], respectively, with both genes showing high expression levels in the liver and intestine of adult zebrafish. The remaining *CYP3C* genes demonstrated rather variable levels of expression in the zebrafish gastrointestinal system [34]. Since our study indicated that BOMR was metabolized by Baculosomes^®^ expressing human CYP3A4 and, as most of the *CYP3* genes are expressed in the zebrafish liver, one could assume that the CYP3 family also contributes to the metabolism of BOMR in ZLM. However, caution is required since our study showed that BOMR was clearly metabolized by recombinant zebrafish CYP1A, which was in line with the results for the human recombinant CYPs as BOMR was predominantly metabolized by recombinant human CYP1A2 and CYP2B6 and only to a lesser extent by recombinant human CYP3A4. Our results are also in accordance with an earlier study in which another substrate of human CYP3A4, i.e., 17β-estradiol, was clearly metabolized by recombinant zebrafish CYP1s [32]. Moreover, we found that ZLM were not able to metabolize the Luciferin-IPA, which is a highly specific substrate for human CYP3A4 [36,37]. A possible explanation for the latter finding is a difference in structure between the active site of human and zebrafish CYP3A enzymes resulting in different drug-metabolizing capacities [41]. This hypothesis is supported by the research of Goldstone et al. [17] who found that the *CYP3A65* gene was identical to human *CYP3A4* for only 54%. Furthermore, concerning the CYP3C family, a study of Corley-Smith et al. [22] revealed that the amino acid sequence of CYP3C1 was only 44%–49% similar to the mammalian CYP3A. Another explanation could be differences in the evolutionary tree as zebrafish *CYP3A* genes do not phylogenetically cluster with mammalian *CYP3A* genes, resulting in different functions of the corresponding enzymes [18,19]. Besides the incapability of ZLM to biotransform a CYP3A4-specific substrate, ZLM and HLM also showed different inhibition profiles when co-incubating BOMR and ketoconazole. This dissimilarity may be due to species-differences in CYP isoforms that interact with BOMR and/or with ketoconazole. Indeed, inhibition by ketoconazole has already been described as being species-specific and concentration-dependent [42,43]. In humans, ketoconazole acts as a non-specific, but potent mixed competitive-noncompetitive inactivator of CYP3A [38]. This observation was confirmed in the present study by the inhibition of the biotransformation of BOMR in CYP3A4 BAC. In contrast to ketoconazole, co-incubation of BOMR with CYP3A4-specific CYP3cide did not inhibit the velocity of resorufin formation by ZLM. Moreover, inhibition of BOMR metabolism by CYP3cide in HLM was less pronounced compared with inhibition by ketoconazole. These findings imply that more than one CYP isoform is involved in the biotransformation of BOMR in zebrafish and humans as evidenced by the reaction phenotyping experiments with recombinant zebrafish CYPs and CYP Baculosomes^®^, respectively. Based upon the results above, we can conclude that BOMR, which was supposed to be metabolized predominantly by human CYP3A4 [44,45], is a non-specific CYP substrate in humans and in zebrafish. In other studies, similar conclusions were drawn for 7-benzyloxy-4-(trifluoromethyl)-coumarin (BFC), which was assumed to be specific for mammalian CYP3A, but appeared to be metabolized by bacterially expressed CYP3A and CYP1 from zebrafish [32,33]. In brief, our research showed that the adult zebrafish liver possesses CYP activity required to metabolize the non-specific CYP substrate BOMR. Regarding the potential contribution of CYP3A65 and/or CYP3C isoforms in BOMR biotransformation, our study remains inconclusive mainly due to a lack of recombinant enzymes of these isoforms.

Zebrafish embryos, however, were not capable of metabolizing BOMR before 72 hpf. Taking the LLOD and LLOQ parameters into account, a small amount of resorufin could be detected at the end of organogenesis, i.e., at 72 and 96 hpf, but the values were still negligible compared to resorufin formation by ZLM. This is not surprising as these time points coincide with major development of the liver and intestine, two pivotal drug-metabolizing organs. The liver gets vascularized around 72 hpf, and reaches its adult configuration around 96 hpf [46,47]. For the intestine, the lumen and epithelium develop craniocaudally between 54 and 102 hpf [48]. These morphological data were further substantiated by a whole-mount in situ hybridization study in which *CYP3A65* mRNA was detected in the liver at 72 hpf, followed by expression of the gene in the intestine at 84 and 96 hpf [35]. Our data are also in accordance with earlier in vitro and in vivo studies on CYP1A activity. Indeed, in vitro assessment of basal CYP1A activity in zebrafish embryos and larvae using 7-ethoxyresorufin-*O*-deethylase (EROD) also demonstrated low levels of metabolite formation around 72 and 96 hpf, with even lower levels around 120 hpf [29,30]. Even after CYP induction, activity levels remained low and a similar temporal trend was observed for basal versus induced CYP1 activity [20,29,31]. In vivo CYP1A activity first appeared in the liver primordium around 56 hpf, followed by the intestine at approximately 80 hpf and reaching a peak at 104 hpf [29]. Furthermore, Alderton et al. [49] demonstrated that zebrafish larvae of three and seven days post-fertilization (dpf) were able to metabolize human CYP probe substrates and drugs, albeit to a small extent. Therefore, these authors postulated that the low metabolite concentrations are unlikely to contribute to the malformations in developmental toxicity studies [49]. In addition, Chng and colleagues [21] showed that zebrafish larvae of 120 hpf produced only two CYP metabolites of testosterone compared with the formation of multiple CYP metabolites in liver microsomes from adult zebrafish. Similar to our findings, these authors thus suggested a difference in function or expression of drug-metabolizing enzymes between larval and adult zebrafish [21]. Finally, in contrast to the in vitro CYP1A activity that has been reported for the whole zebrafish embryo as early as at five hpf [30] and at eight hpf [29], BOMR was not metabolized by these earliest embryonic stages in our study. The presence of CYP activity in the early zebrafish embryo before its basic body plan has been established, is maternally derived [17] and disappears quickly. Since BOMR is a non-specific CYP substrate and clear spatio-temporal differences in CYP isoform expression have been reported [17], this could explain the lack of maternally derived CYP activity in our study.

## 4. Conclusions

In conclusion, this in vitro study demonstrated that the non-specific CYP substrate BOMR was metabolized by adult zebrafish liver microsomes as well as by human liver microsomes. In contrast to the adults, zebrafish embryos were not capable of metabolizing BOMR during a major part of organogenesis. In most teratogenicity assays with zebrafish embryos, this would not be an issue since toxicity is mainly caused by the parent compound itself. However, in case of proteratogenic compounds, which require biotransformation to exert their teratogenic potential, false negative results can occur if the drug-metabolizing capacity in the zebrafish embryo is lacking. Hence, our study indicates that zebrafish embryos have a poor CYP-related metabolizing capacity during organogenesis, which needs to be considered in regards to their use as an alternative animal model in developmental toxicity studies. This information needs to be further strengthened by using other CYP substrates and investigating other phase I reactions and the role of phase II enzymes as well.

## 5. Materials and Methods

### 5.1. Fish Maintenance and Breeding

Adult zebrafish (*Danio rerio*, in house wild-type AB zebrafish line) were housed in glass aquaria of 60 L with filtration system at a density of <1 fish/L. Fish were kept in reverse osmosis water to which commercial sea salts (Instant Ocean^®^ Sea Salt, Blacksburg, VA, USA) and sodium bicarbonate (VWR, Leuven, Belgium) were added in order to obtain pH and conductivity values of 7.5 ± 0.3 and 500 ± 40 µS/cm, respectively. The water temperature was set to 28 ± 1 °C and the fish were subjected to an automated light-dark cycle of 14/10 h. Water parameters and fish health were checked daily and water was renewed once in a fortnight to keep the levels of ammonia (NH_3_), nitrite (NO_2_^−^) and nitrate (NO_3_^−^) below the detection limits, i.e., NH_3_ < 0.02 mg/L, NO_2_^−^ < 0.3 mg/L and NO_3_^−^ ≤ 12.5 mg/L. Fish were fed twice daily with thawed food—alternating *Artemia nauplii*, *Daphnia* and Chironomidae larvae (Aqua Mila, Deurne-Diest, Belgium)—and once daily with granulated food (sturgeon food Duvo^+^, Laroy Group™, Wondelgem, Belgium) [5].

For the collection of zebrafish embryos, adult fish were transferred to a spawning tank the day before mating. The next morning, eggs were collected 45 min after the light was turned on. Subsequently, feces and coagulated eggs were removed by washing the embryos in freshly prepared egg water—same composition as the adult fish medium—with pH and conductivity set to 7.5 and 480 µS/cm, respectively [5]. The zebrafish embryos were kept in egg water using a density of 1 embryo/mL and under the same environmental conditions of light and temperature as for the adults. Dead embryos were removed daily and egg water was renewed every 48 h. The zebrafish embryos were raised until they reached the desired developmental stage.

### 5.2. Tissue Sampling

#### 5.2.1. Adult Zebrafish

Only one sex was used for liver collection as hepatic CYP1A activity was shown to be independent of gender in zebrafish [30]. Six batches of adult female zebrafish between six months and one year of age were utilized for the preparation of zebrafish liver microsomes. Batch 1 and Batch 2 were used for the optimization of the activity assays as well as for the inhibition studies, for which an adequate amount of microsomal protein is needed. Hence, Batch 1 and 2 consisted of 65 and 100 individuals per batch, respectively, whereas the remaining batches (3–6) consisted of 10 animals per batch. After a food deprivation period of 48 h, the fish were euthanized by rapid destruction of the brain and decapitation [50]. The gastrointestinal system was carefully removed from the zebrafish body, followed by identification and isolation of the liver. In order to prevent bile contamination, the gall bladder was carefully discarded. During the dissection process, livers were rinsed with pre-cooled washing buffer (10 mM potassium phosphate (KPO_4_) buffer (BD Gentest™, Woburn, MA, USA) containing 1.15% potassium chloride (KCl) (Analar Normapur^®^, VWR, Leuven, Belgium) at pH 7.4). Liver samples were immediately snap-frozen in liquid nitrogen and stored at −80 °C until the isolation of ZLM. The animal protocols applied in this study were evaluated and approved by the Ethical Committee of Animal Experimentation from the University of Antwerp (Antwerp, Belgium) (ECD 2015-49; 18 September 2015).

#### 5.2.2. Zebrafish Embryos

For each developmental stage—i.e., 5, 24, 48, 72, 96 and 120 hpf—three batches of zebrafish embryos were used and each batch consisted of approximately 2500 embryos. When they reached the desired developmental stage, the embryos were snap-frozen in liquid nitrogen and stored at −80 °C to be used for microsomal protein preparation. 

### 5.3. Isolation of Microsomes

#### 5.3.1. Adult Zebrafish

The protocol for the isolation of ZLM is based on the one described by Hill [51] regarding the preparation of rat liver microsomes. All homogenization steps were performed on ice. Prior to homogenization, liver samples were thawed on ice, weighed and washed with pre-cooled homogenization buffer (10 mM KPO_4_ buffer containing 1.15% KCl, 1 mM ethylenediaminetetraacetic acid (EDTA) and one unit of Halt™ Protease Inhibitor Single-Use Cocktail per 10 mL buffer (the latter two were purchased from Thermo Fisher Scientific, Waltham, MA, USA) at pH 7.4) in order to remove possible remnants of hemoglobin. Subsequently, for each gram of liver tissue, a twofold volume in a milliliter of homogenization buffer was added. The tissue was then homogenized manually in a glass tube by means of a Potter-Elvehjem PTFE pestle. As a final homogenization step, samples were subjected to ultrasonication for (5 × 5) s with intervals of 10 s and an amplitude of 75% using an Ultrasonic Processor VCX 130 (Sonics & Materials Inc., Newton, CT, USA). The homogenate was centrifuged at 12,000× *g* for 20 min at 4 °C, using a Heraeus™ Multifuge™ X3R Centrifuge (Thermo Fisher Scientific). In order to remove the fat layer that had been accumulated on the surface of the resulting supernatant, an additional centrifugation step was performed at 12,000× *g* for 10 min at 4 °C. The purified supernatant—containing the S9-fraction—was then subjected to ultracentrifugation at 100,000× *g* for 60 min at 4 °C, using an Optima™ MAX-XP ultracentrifuge (Beckman Coulter, Indianapolis, IN, USA). The resulting pellet was resuspended in homogenization buffer followed by a second ultracentrifugation step at 100,000× *g* for 40 min at 4 °C. Finally, the resulting microsomal pellet was resuspended in storage buffer (100 mM KPO_4_ buffer containing 250 mM sucrose (Sigma–Aldrich, St. Louis, MO, USA), 1 mM EDTA and 1 unit of Halt™ Protease Inhibitor Single-Use Cocktail per 10 mL buffer), aliquoted and stored at −80 °C until further use. The microsomal protein concentration of the ZLM was determined by means of the microplate procedure of the Pierce™ BCA Protein Assay Kit with bovine serum albumin as a standard (Thermo Fisher Scientific).

#### 5.3.2. Zebrafish Embryos

The ZEM were isolated according to the same protocol as described for the adults. However, a few changes, which will be outlined in the current section, were made. First, during the homogenization of the embryos, no washing steps were performed since contamination with hemoglobin was considered negligible. Second, an additional centrifugation step at 12,000× *g* for 10 min at 4 °C was carried out to remove the high load of melanophores that had been accumulated in the supernatant. 

### 5.4. Benzyloxy-Methyl-Resorufin Assay in Adult Zebrafish Liver Microsomes

The fluorogenic substrate benzyloxy-methyl-resorufin (Vivid^®^ BOMR Substrate, P2865, Thermo Fisher Scientific) was used in order to assess CYP activity in ZLM. According to the Vivid^®^ CYP450 Screening Kits User Guide (2012), BOMR is predominantly metabolized by human CYP3A4. Prior to activity assessment, assay conditions were optimized for substrate and microsomal protein concentration by testing a range of six protein concentrations of ZLM (12.5–400 µg/mL) and seven concentrations of BOMR (0.15–9.6 µM). The optimal microsomal protein concentration and optimal substrate concentration was 200 µg/mL and 1.2 µM, respectively, both values being situated within the linear part of the reaction curve. All CYP activity assays were performed in non-binding black polystyrene 96-well microplates with flat bottom and chimney wells (655900, Greiner Bio-One International GmbH, Kremsmünster, Austria). Positive and negative controls were included in each assay and were subjected to the same protein and substrate concentrations as for the ZLM. Pooled human liver microsomes (Gibco™, HMMCPL–PL050B, Thermo Fisher Scientific) and CYP3A4 Baculosomes^®^ Plus Reagent rHuman (P2377, Thermo Fisher Scientific) were utilized as positive control. Insect Cell Control Supersomes™ (456201, Corning Incorporated, Corning, NY, USA), lacking CYP enzymes, were chosen as negative control. A total incubation volume of 100 µL/well was used. The microsomal reaction was initiated in each well by the addition of substrate solution containing 1.2 µM BOMR, 0.1 mM NADP^+^ (Vivid^®^ NADP^+^, P2879, Thermo Fisher Scientific), 3.33 mM glucose-6-phosphate, 0.3 U/mL glucose-6-phosphate dehydrogenase (Vivid^®^ Regeneration System, P2878, Thermo Fisher Scientific) and 100 mM KPO_4_ buffer (pH 7.4) to the microsomal solution containing 20 µg/100 µL microsomal protein and 100 mM KPO_4_ buffer (pH 7.4). Subsequently, fluorescence was measured for 60 min with 2-min intervals using a Tecan Infinite^®^ 200 PRO microplate reader (Tecan Group Ltd., Männedorf, Switzerland) at λ_ex_ 550 nm and λ_em_ 590 nm. During measurement, the temperature was kept at 28 °C which is within the zebrafish’s optimal water temperature range of 26–28.5 °C [5]. The same temperature was utilized for the controls as, in a previous literature report [30], similar CYP activities could be detected for HLM at 28.5 °C and 37 °C, respectively. The concentration of resorufin (nM)—a metabolite of BOMR—produced at each time point was determined from a standard curve that had been established by using the pure fluorescent metabolite (Vivid^®^ Red Fluorescent Standard, P2874, Thermo Fisher Scientific). The average values of the negative control were subtracted from the individual result values obtained for ZLM, HLM and CYP3A4 BAC. Reaction velocities were calculated in units of picomoles of resorufin formed per minute per milligram of microsomal protein (pmol/min/mg MP). The lower limit of detection (LLOD) and the lower limit of quantification (LLOQ) were 3.41 nM (0.17 pmol/min/mg MP) and 7.58 nM (0.39 pmol/min/mg MP), respectively. For each batch of ZLM, three technical replicates of the activity assay were performed.

### 5.5. Benzyloxy-Methyl-Resorufin Assay in Microsomes from Whole Zebrafish Embryo Homogenates

Similar to the adult zebrafish, the fluorogenic substrate BOMR was used in order to assess the capacity of biotransformation in zebrafish embryos from 5 to 120 hpf. Pooled human liver microsomes (Gibco™, HMMCPL–PL050B, Thermo Fisher Scientific) and CYP3A4 Baculosomes^®^ Plus Reagent rHuman (P2377, Thermo Fisher Scientific) were utilized as positive control and Insect Cell Control Supersomes™ (456201, Corning Incorporated) were chosen as negative control. Additionally, ZLM of Batch 1 were included to be used as a reference for the different developmental stages. Therefore, the activity assays with ZEM were performed according to the same protocol and at the same microsomal protein (200 µg/mL) and substrate (1.2 µM) concentration as described for the experiments using ZLM. At the end of the assay, fluorescence was measured for 60 min with 2-min intervals at 28 °C using a Tecan Infinite^®^ 200 PRO microplate reader (Tecan Group Ltd.) at λ_ex_ 550 nm and λ_em_ 590 nm. Resorufin concentration and reaction velocities (pmol/min/mg MP) were calculated in a similar way as for the ZLM. The average values of the negative control were subtracted from the individual result values obtained for the ZEM, ZLM, HLM and CYP3A4 BAC. For each batch of ZEM, three technical replicates of the activity assay were performed.

### 5.6. Inhibition Studies with Adult Zebrafish Liver Microsomes

#### 5.6.1. Ketoconazole

Inhibition studies in ZLM were performed by co-incubation of the BOMR substrate with ketoconazole (K1003, Sigma–Aldrich), which is known to be a non-specific but potent inhibitor of human CYP3A. As inhibition studies require an adequate amount of microsomal protein, Batch 1 and Batch 2 of ZLM were used due to their large sample size. Positive and negative controls were similar to those used in the activity assays with BOMR. Since inhibition studies show higher sensitivity when performed at low substrate and microsomal protein concentrations [38,43], the latter were set to 0.8 µM BOMR and 100 µg/mL microsomal protein, both within the linear part of the reaction curve. Inhibition assays were executed in non-binding black polystyrene 96-well microplates with flat bottom and chimney wells (655900, Greiner Bio-One International GmbH) with a total incubation volume of 100 µL/well. A range of eight ketoconazole concentrations (0–20 µM) was pre-incubated with 10 µg/100 µL microsomal protein diluted in 100 mM KPO_4_ buffer (pH 7.4) for 10 min. Subsequently, the microsomal reaction was initiated in each well by the addition of 0.8 µM BOMR, 0.1 mM NADP^+^ (Vivid^®^ NADP^+^, P2879, Thermo Fisher Scientific), 3.33 mM glucose-6-phosphate and 0.3 U/mL glucose-6-phosphate dehydrogenase (Vivid^®^ Regeneration System, P2878, Thermo Fisher Scientific) diluted in 100 mM KPO_4_ buffer (pH 7.4) to the pre-incubated mixture. Finally, measurements of fluorescence and calculations of resorufin concentrations and reaction velocities were executed the same way as for the activity assays with BOMR. Additionally, IC_50_ values—concentrations of ketoconazole to cause 50% inhibition of original CYP activity—were determined for ZLM as well as for the positive controls. For each batch of ZLM, two technical replicates of the inhibition assays were performed.

#### 5.6.2. CYP3cide

Inhibition studies with CYP3cide (PZ0195, Sigma–Aldrich)—a mechanism-based and CYP3A4-specific inhibitor [39]—were carried out for ZLM of Batch 1 and Batch 2. The assays with CYP3cide were performed according to the same protocol as described for ketoconazole. However, a few adjustments were made. First, two separate ranges of CYP3cide concentrations (0–2 µM and 0–4 µM) were used. Second, as biotransformation of CYP3cide is required to exert its inhibitory potential, an NADPH-regenerating system containing 1.3 mM NADP^+^, 3.3 mM glucose-6-phosphate, 0.4 U/mL glucose-6-phosphate dehydrogenase and 3.3 mM magnesium chloride (451220 and 451200, Corning Incorporated) was added to the pre-incubated mixture. The microsomal reaction was then initiated by the addition of BOMR diluted in 100 mM KPO_4_ buffer (pH 7.4). IC_50_ values of CYP3cide were calculated for ZLM and positive controls. For Batch 1 and Batch 2 of ZLM, two technical replicates of the CYP3cide assays were performed.

### 5.7. Benzyloxy-Methyl-Resorufin Assay in CYP Baculosomes^®^

In these activity assays, CYP1A2, CYP2B6, CYP2C9, CYP2C19, CYP2D6 and CYP3A4 Baculosomes^®^ Plus Reagent, rHuman (P2792, P3028, P2378, P2570, P2283 and P2377, respectively, Thermo Fisher Scientific) were used. CYP Baculosomes^®^ are microsomes prepared from insect cells transfected with cDNA encoding for the above-mentioned CYP isoforms. A BOMR concentration of 3 µM was applied as recommended by the Vivid^®^ CYP450 Screening Kits User Guide (Life Technologies™, Thermo Fisher Scientific). For all CYP Baculosomes^®^, a microsomal protein concentration of 35 µg/mL was utilized, which represents the mean of the protein concentrations advised for the six different CYP Baculosomes^®^. Insect Cell Control Supersomes™ (456201, Corning Incorporated) were included as negative control and were subjected to the same protein and substrate concentrations as for the CYP Baculosomes^®^. A total incubation volume of 100 µL/well was used. The microsomal reaction was initiated in each well according to the same protocol as described for the BOMR assay with ZLM. Fluorescence was measured in a similar way as for the ZLM, except for the temperature that was kept at 37 °C (human body temperature) since this was recommended by the manufacturer. The concentration of resorufin produced at each time point was determined from a standard curve that had been established by using the pure fluorescent metabolite (Vivid^®^ Red Fluorescent Standard, P2874, Thermo Fisher Scientific). The average values of the negative control were subtracted from the individual result values obtained for the different CYP Baculosomes^®^. The velocities of resorufin formation were calculated in pmol/min/pmol CYP as the specific CYP content per milligram of total protein was different between the CYP Baculosomes^®^ (the specific CYP content was mentioned in the data sheet for each batch of CYP Baculosomes^®^, i.e., 91, 170, 480, 213, 100 and 313 pmol/mg of total protein for CYP1A2, 2B6, 2C9, 2C19, 2D6 and 3A4, respectively). The LLOD and LLOQ were determined and denoted as pmol/min/mg MP, i.e., 0.54 pmol/min/mg MP and 1.60 pmol/min/mg MP, respectively. For each of the six CYP Baculosomes^®^, three technical replicates of the BOMR assays were performed.

### 5.8. Benzyloxy-Methyl-Resorufin Assay in Recombinant Zebrafish CYPs

Zebrafish CYP1A, CYP1B, CYP1C1, CYP1C2 and CYP1D were cloned and co-expressed with human cytochrome P450 reductase in *Escherichia coli* (JM109) and purified, all according to Scornaienchi et al. [32]. Briefly, each *CYP* gene was cloned with the *omp*A2+ leader sequence, which targets the expressed CYPs to the bacterial outer membrane. The *omp*A2+ sequence is excised after the protein is inserted into the membrane, thereby allowing the expression of the full-length CYP protein [32]. Each *CYP* gene/*omp*A2+ sequence was ligated into a pCW vector, and co-transfected with the human NADPH-CYP reductase ligated into a pACYC vector [32]. Overnight cultures were treated with ampicillin (50 µg/mL) and chloramphenicol (25 µg/mL) (Thermo Fisher Scientific), while isopropyl β-d-1-thiogalactopyranoside (1 mM, Thermo Fisher Scientific) was added when cultures had reached an OD_600_ between 0.7 and 1.0. Expression of each CYP was optimized with the addition of 0.1 to 1 mM δ-aminolevulinic acid (MP Biomedicals, Santa Ana, CA, USA). Cells were allowed to express the CYP proteins for 20–24 h, after which time they were harvested and the bacterial membranes were purified. Total protein was determined for each CYP stock using a bicinchoninic acid assay kit (Thermo Fisher Scientific).

Resorufin generation from BOMR was determined for each of the zebrafish CYPs. The volume of all the reactions was 50 µL, which were performed in black 384-well plates (Thermo Fisher Scientific). Enzyme buffer consisted of 50 mM TrisHCl and 100 mM NaCl (Thermo Fisher Scientific) adjusted to pH 7.8. NADPH (Sigma–Aldrich) was prepared daily, and the final NADPH concentration in each reaction was 150 µM. Total protein concentrations ranged between 2 and 2.5 µg/50 µL reaction for the different CYPs. Fluorescence was measured in the CYP reactions for 8 min at 1-minute intervals using a BioTek Synergy2 microplate reader (BioTek U.S., Winooski, VT, USA) at λ_ex_ 540 nm and λ_em_ 590 nm. Reactions were held at 29 °C. For the first experiment, eight BOMR concentrations (0.055–40 µM, plus a 0 µM control-dimethyl sulfoxide only) were tested in duplicate wells. The second experiment was conducted at 1.5 µM BOMR in triplicate wells, given that resorufin generation rates decreased at BOMR concentrations above this level for CYP1A, CYP1B and CYP1C2. The LLOD and LLOQ for the zebrafish CYPs were 0.0008 pmol/min/µg total protein, and 0.0023 pmol/min/µg total protein, respectively.

### 5.9. Luciferin-IPA Assay with Adult Zebrafish Liver Microsomes

This activity assay was performed with Luciferin-IPA (P450–Glo™ CYP3A4 Assay, V9001, Promega Corporation, Madison, WI, USA), which is a highly specific luminogenic substrate for human CYP3A4 [36,37]. Pooled human liver microsomes (Gibco™, HMMCPL–PL050B, Thermo Fisher Scientific) and CYP3A4 Baculosomes^®^ Plus Reagent rHuman (P2377, Thermo Fisher Scientific) were utilized as positive control and Insect Cell Control Supersomes™ (456201, Corning Incorporated) were used as negative control. For ZLM (Batch 1 and 2) and HLM, the optimal microsomal protein concentration and optimal substrate concentration was determined by testing a range of five protein concentrations (25–400 µg/mL) and a range of six Luciferin-IPA concentrations (1–32 µM), respectively. Since metabolite concentrations for ZLM were below the LLOQ, the optimal microsomal protein concentration (200 µg/mL) and the optimal substrate concentration (4 µM) obtained for HLM were applied in the Luciferin-IPA assays. All assays were performed in non-treated Nunc™ F96 Microwell™ white polystyrene plates (236205, Thermo Fisher Scientific) with a total incubation volume of 50 µL/well. Prior to the initiation of the microsomal reaction, 10 µg/50 µL microsomal protein diluted in 100 mM KPO_4_ buffer (pH 7.4) was pre-incubated with 4 µM Luciferin-IPA substrate for 10 min. Subsequently, the microsomal reaction was initiated in each well by the addition of NADPH-regenerating system containing 1.3 mM NADP^+^, 3.3 mM glucose-6-phosphate, 0.4 U/mL glucose-6-phosphate dehydrogenase and 3.3 mM magnesium chloride (451220 and 451200, Corning Incorporated) in 100 mM KPO_4_ buffer (pH 7.4) to the pre-incubation mixture. The final reaction mixture was then incubated for 10 min at 37 °C (optimal temperature for HLM) followed by the addition of 50 µL of Luciferin Detection Reagent diluted in Reconstitution Buffer (V859A and V144A, Promega Corporation) to each microplate well to stop the microsomal reaction. Consequently, a luminescent signal was initiated. Subsequently, the luminescent signal was stabilized by incubation of the mixture for 20 min at room temperature. Finally, luminescence was measured using a Tecan Infinite^®^ 200 PRO microplate reader (Tecan Group Ltd.). The concentration of D-Luciferin (metabolite of Luciferin-IPA) was determined by comparing luminescence from the microsomal reactions to that from a d-Luciferin standard curve (Beetle Luciferin, Potassium Salt, E1601, Promega Corporation). For the positive controls as well as for the ZLM, the average values of the negative control were subtracted from the individual result values. Reaction velocities were calculated in pmol/min/mg MP and the LLOD and LLOQ were 0.77 nM (0.38 pmol/min/mg MP) and 1.74 nM (0.87 pmol/min/mg MP), respectively. For each batch of ZLM, three technical replicates of the activity assays were performed.

### 5.10. Mathematical and Statistical Analyses

For all activity and inhibition assays, reaction velocities were calculated within the linear part of the reaction curve. LLOD and LLOQ were determined as described by Şengül [52]. Optimal substrate concentrations were determined by nonlinear regression analysis using the substrate inhibition model in GraphPad Prism (version 6.05; GraphPad Software, Inc., La Jolla, CA, USA). Calculation of reaction velocities was performed in Microsoft Excel^®^ 2010 (Microsoft Corporation, Redmond, WA, USA). The results from the CYP activity assays with BOMR were statistically analyzed using IBM SPSS Statistics (version 23; IBM, Armonk, NY, USA). A nonparametric Levene’s test was used to test homogeneity of variances for EM of 72 hpf and 96 hpf and for ZLM. Subsequently, the results for these age groups were subjected to a Kruskal–Wallis test, followed by pairwise comparisons (Mann–Whitney test) to detect differences between the groups. Differences were considered statistically significant when *p* ≤ 0.05. Estimation of IC_50_ values was performed by a nonlinear regression analysis with a four-parameter logistic curve in GraphPad Prism (version 6.05; GraphPad Software, Inc.).

## Figures and Tables

**Table 1:** Most important drug-metabolizing cytochrome P450 (CYP) enzymes in man: relative abundance in human liver and contribution to oxidative biotransformation of drugs (reviewed in [15,40]).

CYP Isoform	Content in Liver (% of Total CYP)	% of Drugs Metabolized by CYP
CYP3A4/5	±30	±50
CYP2D6	±4	±30
CYP2B6	2–10	±25
CYP2C8, -2C9, -2C19	±20	±16
CYP1A2	±13	±4

**Table 2:** Overview of resorufin formation by CYP Baculosomes^®^ (BAC) with 3 µM BOMR and by recombinant zebrafish CYPs with 1.5 µM BOMR.

Recombinant CYPs	Resorufin Formation
**CYP Baculosomes^®^^1^**	**pmol/min/pmol CYP**
CYP1A2 BAC^®^	0.519 ± 0.100
CYP2B6 BAC^®^	0.497 ± 0.133
CYP3A4 BAC^®^	0.133 ± 0.070
CYP2C9 BAC^®^	0.026 ± 0.002
CYP2C19 BAC^®^	<LLOQ
CYP2D6 BAC^®^	<LLOQ
**Recombinant Zebrafish CYPs ^1^**	**pmol/min/µg Total Protein**
CYP1A	1.152 ± 0.068
CYP1B	0.105 ± 0.008
CYP1C2	0.078 ± 0.011
CYP1C1	0.004 ± 0.001
CYP1D	<LLOQ

^1^ Mean value of three technical replicates ± standard deviation. LLOQ, lower limit of quantification; BOMR, benzyloxy-methyl-resorufin; CYP, cytochrome P450.
